# Physical exercise recommendations for patients with polycythemia vera based on preferences identified in a large international patient survey study of the East German Study Group for Hematology and Oncology (OSHO #97)

**DOI:** 10.1002/cam4.6413

**Published:** 2023-08-09

**Authors:** Sabine Felser, Julia Rogahn, Lina Hollenbach, Julia Gruen, Philipp le Coutre, Haifa Kathrin Al‐Ali, Susann Schulze, Lars‐Olof Muegge, Veronika Kraze‐Kliebhahn, Christian Junghanss

**Affiliations:** ^1^ Department of Internal Medicine, Clinic III—Hematology, Oncology and Palliative Care Rostock University Medical Center Rostock Germany; ^2^ Department of Hematology, Oncology, and Cancer Immunology, Campus Virchow‐Klinikum Charité‐ Universitätsmedizin Berlin Berlin Germany; ^3^ Krukenberg Cancer Center Halle University Hospital Halle Halle (Saale) Germany; ^4^ Department of Internal Medicine, Medical Clinic II Carl‐von‐Basedow‐Klinikum Merseburg Germany; ^5^ Department of Internal Medicine III Heinrich Braun Klinikum Zwickau Zwickau Germany; ^6^ MPN‐Netzwerk e. V. Bonn Germany

**Keywords:** exercise recommendations, fatigue, myeloproliferative neoplasms, physical activity preferences, polycythemia vera, quality of life

## Abstract

**Background:**

Exercise therapy during cancer treatment reduces symptom burden and improves quality of life (QoL). Polycythemia vera (PV) is a myeloproliferative neoplasia associated with good overall survival (up to decades) but a significant symptom burden, including thromboembolic events and dysesthesias. There are no specific exercise recommendations for patients with PV. Thus, we aimed to determine the exercise preferences of patients with PV and to derive specific recommendations based on the most commonly reported symptoms.

**Methods:**

This multicenter survey included patients with PV ≥18 years old. Demographic, clinical, and disease burden data were collected. The severity of selected symptoms was assessed using the adapted Myeloproliferative Neoplasms Symptom Assessment Form: 0 (absent), 1–30 (mild), 31–70 (moderate), or 71–100 (severe). The patients' information needs about physical activity (PA) and exercise preferences were recorded depending on their motivation and analyzed with regard to demographic aspects.

**Results:**

The sample comprised 182 patients (68% female, 61 ± 12 years). The prevalence of moderate‐to‐severe symptoms was 60% for fatigue, 44% for concentration problems, and 35% for bone/muscle pain. Other commonly reported symptoms included skin reactions (49%), splenomegaly (35%), and increased bleeding tendency (28%). Overall, 67% of respondents requested more information regarding PA. Patients with PV preferred individual training (79%) located outdoors (79%) or at home (56%). Regarding the amount of training, sports‐inactive patients preferred a frequency of 1–2 times/week and session durations of 15–45 min, whereas sports‐active patients preferred 3–4 times/week and 30–60 min (*p* < 0.001). Higher sport‐inactiveness was observed in patients with lower educational level compared to patients with higher educational level (69% vs. 50%, *p* = 0.021). For beginners, combined resistance‐endurance (circuit) training two times/week, which can be performed outdoors or at home, should be recommended. In the case of splenomegaly or bleeding symptoms, exercises with a low injury risk should be chosen.

**Conclusion:**

PA is important for patients with PV; therefore, counseling should be integrated into the treatment plan. Specifically, patients with low educational level should be addressed. Prospective studies are warranted to evaluate the effects of the novel exercise recommendations.

## 1 BACKGROUND

Physical activity (PA) is a complementary, non‐pharmacological way of alleviating symptoms and improving physical function and quality of life (QoL). Several studies have shown that exercise therapy decreases mortality in patients with cancer.^1^, ^2^, ^3^, ^4^, ^5^ The development of disease‐specific exercise recommendations is based on the disease‐ and therapy‐related side effects as well as the symptom burden. The current guidelines regarding exercise in patients with cancer mainly refer to entities with high incidence rates, such as prostate and breast carcinoma or hematological diseases such as lymphoma or leukemia.^6^, ^7^, ^8^

Currently, there are no specific evidence‐based recommendations for patients with polycythemia vera (PV) nor for patients with other myeloproliferative neoplasms (MPN). In Europe, the incidence of PV ranges from 0.4 to 2.8 per 100,000 people.^9^ The disease course often occurs over decades and the median overall survival for low‐risk patients is <20 years.^10^ In addition, due to improved diagnostic methods, PV is diagnosed earlier. Combined with the current treatment options, this leads to a chronic disease with a complex burden and reduced QoL.^11^, ^12^, ^13^, ^14^ Typically, the clinical picture is characterized by blood viscosity changes. Patients with PV commonly experience headache, erythromelalgia, as well as other consequences of microcirculatory disorders such as cerebrovascular, cardiovascular, and thromboembolic complications or hemorrhagic diathesis.^15^ In addition, patients experience typical symptoms such as skin reactions, splenomegaly, or bleeding tendency.^14^

In a large multicenter survey, performed within the framework of the East German Study Group for Hematology and Oncology, we analyzed the exercise behavior of patients with MPN.^16^ The results showed, among other things, that especially patients with PV reduce their PA in everyday life as well as in sports due to the disease and the associated disease burden. In addition, sports‐inactive patients with MPN felt more often insufficiently informed about the importance and possibilities of PA than sports‐active patients. The majority of patients with MPN would like more information about PA. Consequently, we conducted more in‐depth detailed analyses within the PV cohort and data beyond the published^16^ are presented.

We conducted the current study to gain insights into (i) the level of information and request for more information regarding exercise therapy options for patients with PV and (ii) to learn more about exercise preferences. Subsequently, (iii) we developed symptom‐based exercise recommendations for patients with PV.

## 2 METHODS

### 2.1 Study design, participants, and inclusion criteria

The design of the study has been published in detail earlier, see Felser et al.^16^ Briefly, the study was designed as a multicenter cross‐sectional survey. It was approved by the Ethics Committee of the University of Rostock (A2020‐0274) and registered with the German Registry of Clinical Trials (DRKS00023698). Patients ≥18 years with MPN^17^ were eligible to participate in the survey. Patients with MPN from institutions of the East German Hematology and Oncology Study Group (OSHO, Data S1) were asked to participate and complete a hardcopy questionnaire (enrollment from January 2021 to September 2021). From April 2021 to September 2021, the study was amended by an online version of the survey consisting of the same set of questions. The participants included patients of the LeukaNET/Leukemia‐Online patient network as well as the German, Austrian, and Swiss MPN patient network. The data presented here include only patients with PV.

### 2.2 Questionnaire

#### 2.2.1 Demographic data

The self‐administered survey comprised questions about gender, age, weight and height, school education, family, and professional status. The body mass index (BMI) was calculated (body weight [kg]/height [m^2^]) and classified as <18.5, 18.5–24.9, 25.0–29.9, and ≥30.0, representing, underweight, normal weight, overweight, and obesity, respectively.^18^ Years of education (school) were categorized as ≤10 or >10 years.

#### 2.2.2 Clinical data

Age at diagnosis and the current therapies were assessed.

#### 2.2.3 Disease burden

Symptoms such as skin reactions or splenomegaly were surveyed. For some items such as fatigue and concentration problems, an adapted version of the Myeloproliferative Neoplasm Symptom Assessment Form (MPN‐SAF)^19^ was used. Each item was rated from 0 (absent) to 100 (worst imaginable). The symptom severity was divided into four categories: absent (0), mild (1–30), moderate (31–70), and severe (>70).

#### 2.2.4 Information level

The patients' level of information regarding the importance of and opportunities for PA and their need for information about this topic were recorded.

#### 2.2.5 Motivation to participate regularly in sports

The five stages of the transtheoretical model of behavioral change were used to determine the motivation to participate regularly in sports.^20^, ^21^ The questionnaire is provided in Data S2. The answers were dichotomized: not regularly active in sports (Stages 1, 2, and 3: precontemplation, contemplation, and preparation, respectively) or regularly active in sports (Stages 4 and 5: action and maintenance, respectively).

#### 2.2.6 Physical exercise preferences

The patients were asked to indicate their physical exercise preferences: the kind of training, location, frequency, and time per session.

### 2.3 Derivation of the exercise recommendations

To identify symptom‐based exercise recommendations for PV patients the method of *integrative decision making* was chosen.^22^ In the first step, sports scientists and physiotherapists deduced exercise recommendations for patients with PV based on published data (PubMed search). Due to lack of studies on effects of exercise interventions in patients with PV or MPN, primary evidence was deduced from studies of patients with other hematologic neoplasms or solid tumors. If no evidence‐based symptom recommendations for cancer were available, the search was extended to other relevant patient cohorts. The deduced recommendations were supplemented with advices on common symptoms management for PV. To assist PV patients starting sports activities, we identified training possibilities/options considering the patients preferences. Following, the exercise recommendations compiled in step one were presented to oncologists as well as patients from the German, Austrian, and Swiss MPN patient networks. Oncologists and PV patients networks obtained the opportunity to express their subjective opinions or objections to the proposed exercise recommendations. The focus was set on the avoidance of adverse events during or due to training. In the third step, all relevant objections were included to the initial exercise recommendations. Acquiring the consent of all parties involved in the decision process, it was assumed that the new training recommendations are “safe enough to try.”

### 2.4 Statistical analysis

Continuous data are reported as the mean ± standard deviation, and categorical variables are presented as numbers and percentages. Mean differences were tested using the chi‐squared test (Fisher's exact test). All data were analyzed using SPSS (version 25.0, IBM, Armonk, NY, USA). Statistical significance was assumed for *p*‐values <0.05.

## 3 RESULTS

### 3.1 Demographic and clinical data

In total, we received 766 questionnaires, of which 41% (*n* = 315) were completed in the hardcopy format and 59% (*n* = 451) were completed online. Due to missing information regarding diagnosis or too many missing details, we excluded 8% (*n* = 60) of the questionnaires. Thus, we included 706 questionnaires in the analysis. The PV cohort comprised 182 questionnaires, 26% (*n* = 48) in the hardcopy format and 74% (*n* = 134) in the online format. The demographic and clinical data of the patients are presented in Table 1. The analyzed cohort included 68% (*n* = 124) women. The mean age of patients with PV was 61 ± 12 years (median 60 years, range 21–85 years). According to the BMI calculation, 39% (*n* = 70) of the participants were overweight or obese. At the time of the survey, 48% (*n* = 87) of patients were with PV were working, of whom 14% (*n* = 12) were on sick leave. The first diagnosis of PV was a mean of 7.9 ± 7.5 years ago at the time of the survey (diagnosed between 1981 and 2021). The average age at diagnosis was 53 ± 13 years. The most frequently mentioned therapies were phlebotomy and anticoagulation, each reported by 40% (*n* = 72) of the patients, followed by cytostatics (33%, *n* = 60).

**TABLE 1:** Demographics, clinical data, and disease burden (*n* = 182).

Characteristic	*n*	Category	Values
Gender	182	Female Male	124 (68) 58 (32)
Age (years)	182		61 ± 12
BMI (kg/m^2^)	180	<18.5 18.5–24.9 25.0–29.9 ≥30.0	2 (1) 108 (60) 51 (28) 19 (11)
School education (years)	177	≤10 >10	76 (43) 101 (57)
Family status	181	Single Married/living with a partner Other	35 (19) 135 (75) 11 (6)
Professional status	178	Working Retired Other	87 (48) 83 (46) 10 (6)
Age of diagnosis (years)	175		53 ± 13
Time since diagnosis (years)	175		8 ± 7
Current therapy	182	Phlebotomy Anticoagulants Cytostatics Interferon JAK2‐inhibitors Watch and wait Others	72 (40) 72 (40) 60 (33) 42 (23) 39 (21) 27 (15) 3 (2)
Disease burden	178 176 178 181 181 179 176	Skin reactions Splenomegaly Increased bleeding tendency Weight gain Unintentional weight loss Osteoporosis Thrombosis during last 3 months	88 (49) 64 (35) 50 (28) 36 (20) 21 (12) 14 (8) 5 (3)

*Note*: Data are presented as the number of participants (%) for categorical variables and as mean ± standard deviation for continuous variables.

Abbreviations: BMI, body mass index; JAK, Janus kinase; *n*, number of patients.

### 3.2 Disease burden

**FIGURE 1:**
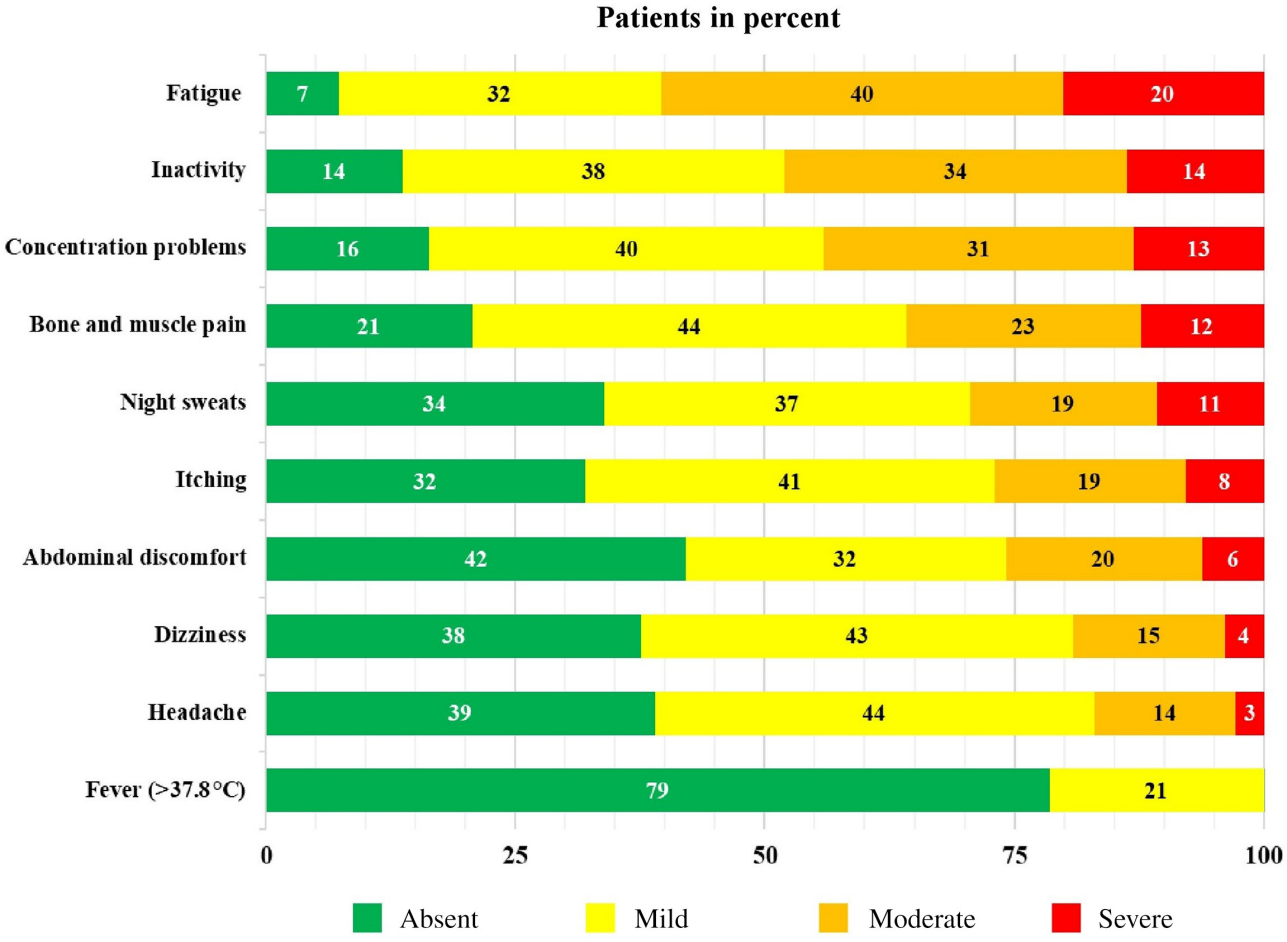
Symptom severity of patients with polycythemia vera (*n* = 212). Surveyed using an adapted version of the Myeloproliferative Neoplasm Symptom Assessment Form (MPN‐SAF).^19^ Each item was scored on a scale from 0 (absent) to 100 (worst imaginable).

The frequencies of typical symptoms are shown in Table 1. The most frequently mentioned symptoms were skin reactions (49%, *n* = 88), splenomegaly (35%, *n* = 64), and increased bleeding tendency (28%, *n* = 50). Figure 1 provides an overview of the severity of selected symptoms. Symptoms with moderate‐to‐severe manifestation included fatigue (60%), inactivity (48%), concentration problems (44%), and bone/muscle pain (35%).

### 3.3 Information level

When asked whether they felt sufficiently informed about the importance and opportunities of PA in regards to their disease, 47% (*n* = 77) of the patients with PV said no. No significant group differences depending on demographic data were observed. Regardless of the level of information, 67% (*n* = 108) of the patients with PV stated they would like to receive more information about PA. The proportion of patients who would like to receive more information about PA was particularly high among patients with high levels of education (>10 years, 77%) and age ≤60 years (73%). However, the difference was not significant compared to patients with low educational level (≤10 years, 60%, *p* = 0.089) or patients >60 years (60%, *p* = 0.133).

### 3.4 Motivation to participate regularly in sports

The question about motivation to participate regularly in sports was answered by 86% (*n* = 157) of the patients with PV. The analysis of the stages of behavioral change revealed that 38% (*n* = 60) of them were not action oriented (precontemplation stage), and 20% (*n* = 31) were in the contemplation or preparation stage. In total, 42% (*n* = 66) patients with PV reported regular participation in sports (action and maintenance stages). The proportion of those who felt adequately informed about the importance of and opportunities for PA was significantly higher among those who were active in sports than among those who were not active in sports (64% vs. 46%, *p* = 0.036).

### 3.5 Physical exercise preferences

Table 2 presents an overview of the physical exercise preferences of the patients with PV. Individual training was preferred by 79% (*n* = 126) of patients with PV, and group training was preferred by 40% (*n* = 64). The preferred training locations were outdoors (79%, *n* = 127), followed by at home (56%, *n* = 89) and an indoor swimming pool (29%, *n* = 47). Group differences of training type or location were found depending on BMI, school education, and professional status. While 64% of normal‐weight PV patients reported preferring training at home, the proportion in overweight/obese patients was 45% (*p* = 0.032). Among the higher educated patients, 88% indicated a preference for individual training. In comparison, among the patients with lower education level this was 69% (*p* = 0.005). Employed PV patients prefered significantly higher individual (85%) and outdoor training (89%) compared to retired patients (71% and 70%, respectively, *p* = 0.051 and *p* = 0.005, respectively).

**TABLE 2:** Physical exercise preferences depending on motivation for regular sports.

Questions	Total cohort	Not active in sports	Active in sports	*p*‐value
	*n* = 160	*n* = 91	*n* = 66	*χ* ^2^‐test
	*n* (%)	(%)	(%)	
Which kind of training would you prefer?^a^				
Individual training	126 (79)	82	80	0.838
Group training	64 (40)	33	46	0.135
Which location would you prefer?^a^				
Outdoor	127 (79)	79	82	0.838
At home	89 (56)	53	62	0.323
Indoor swimming pool	47 (29)	28	35	0.378
Physiotherapy	30 (19)	22	15	0.405
Sports hall	26 (16)	12	23	0.078
Gym	26 (16)	12	21	0.119
Other	12 (8)	–	–	–
What is your favorite training frequency? [times per week]				**<0.001** *
1–2	86 (54)	74	26	
3–4	56 (35)	21	54	
>4	17 (11)	5	20	
Which training time would you prefer? [min]				**<0.001** *
<15	18 (11)	18	1	
15–30	37 (23)	31	14	
30–45	42 (26)	23	32	
45–60	43 (27)	20	35	
>60	19 (12)	8	18	

Abbreviation: *n*, number of patients.

^a^ Multiple response possible.

* *p* < 0.001; bold: statistically significant.

Regarding the amount of training, significant group differences were observed, especially between sports‐active and sports‐inactive patients with PV. Of the sports‐inactive patients with PV, 74% indicated a preference for an exercise frequency of 1–2 times/week, with 3–4 times/week preferred by 21%. In contrast, 26% of the sports‐active patients with PV preferred a frequency of 1–2 times/week and 54% preferred 3–4 times/week (*p* < 0.001). Regarding the training time per session, sports‐inactive patients with PV preferred 15–45 min; in contrast, sports‐active patients with PV preferred 30–60 min (*p* < 0.001). It should be mentioned that the proportion of patients who are sports‐inactive is significantly higher in patients with lower educational level compared to patients with higher educational level (69% vs. 50%, *p* = 0.021). In addition, the analyses showed that patients with lower educational level are more often overweight/obese compared to patients with higher educational level (54% vs. 28%, *p* < 0.001). The proportion of overweight/obese patients among sports‐inactive patients is significantly higher than among sports‐active patients (50% vs. 24%, *p* = 0.002).

### 3.6 Exercise recommendations for patients with polycythemia vera

Table 3 summarizes the training recommendations and advice.

**TABLE 3:** Exercise recommendations and advices for patients with polycythemia vera.

Category of the FITT principle^a^	General recommendations adapted to patients' preferences
Frequency	At least 2 sessions per week
Intensity	Moderate to severe, progressive
Time	30–60 min per session
Type	Combined resistance‐endurance training or separate from each other

Symptoms/side effects	Special recommendations
Fatigue/concentration problems	Yoga, tai‐chi/qigong as an alternative or supplement to combined resistance‐endurance training
Musculoskeletal pain	Emphasis on resistance training or yoga
Anxiety/depression	Emphasis on endurance training or yoga, relaxation
Obesity	Joint‐relieving sports (e.g., swimming, cycling), muscle hypertrophy
Skin reactions	Avoiding direct sunlight (possibly also water), using UV‐protection
Splenomegaly	Equipment training, avoiding contact/ball sports
Increased bleeding tendency	Avoiding sports with a risk of falling and injury
Increased blood viscosity ➔ headache/dizziness	No quick changes of position, training in company, compensation of fluid losses

Preferred kind of training	Special recommendations (examples)
Individual training	Indoor: circuit training with own body weight or small equipment, ergometer training, swimming, aqua jogging Outdoor: circuit training with own body weight and included parts of rapid walking, running, cycling or skipping rope; nordic walking, skiing, rowing
Group training	Indoor: various rehabilitation or fitness courses also aqua jogging/gymnastics Outdoor: walking, running, cycling, or circuit training in company of others

*Note*: Higher age, overweight, fatigue or concentration problems are independent *risk factors for falls*.^23^ Recommendations: (in addition) coordination training incl. balance training and the use of fall prevention strategies (e.g., poles when walking).

^a^ FITT is an acronym for frequency, intensity, time, and type. Adjustments required for (multi)comorbidity; generally medical approval should be obtained before starting exercise.

#### 3.6.1 Motivation

Patients with PV who are not action oriented with regard to regular sports should be motivated to engage in PA of any intensity, accompanied by a reduction in sedentary behavior, in order to reduce the risk of diseases such as heart disease and type 2 diabetes.^24^, ^25^ A high level of PA after diagnosis also reduces the risk of cancer‐specific mortality, as shown in studies of solid tumors, and we assume that the benefits are transferable to other entities.^4^ Increasing PA, also in the form of daily activities, could help patients with PV regulate their body weight. This in turn might have a positive effect on symptom burden^26^ and reduce the risk of falls.^16^, ^23^ In addition, it is known that in patients with MPN, QoL increases with higher activity levels,^27^ and the likelihood of anxiety and depression decreases.^12^

#### 3.6.2 Exercise recommendations

When using targeted movement interventions to alleviate symptoms, patients with PV should focus attention on fatigue and pain. Both symptoms have a high prevalence in patients with PV, limit QoL, and are barriers to PA.^27^, ^28^, ^29^, ^30^ In addition, fatigue is often associated with memory and concentration problems.^31^

Adults with hematological malignancies can reduce both fatigue and depression with moderate‐intensity endurance training alone or combined resistance‐endurance training.^32^ For cancer survivors, 150 min/week of moderate‐to‐vigorous‐intensity exercise is recommended to alleviate fatigue, although there is insufficient evidence for a linear dose response. In contrast, preliminary study data indicate that there may be a dose–response effect in patients with depression.^6^ Huberty et al.^33^, ^34^ showed that lower training volumes also have positive effects in patients with MPN. In their studies, 50 min of yoga training per week had small‐to‐moderate effects on the symptoms of fatigue, pain, anxiety, depression, and sleep disturbance. Based on this finding, tai‐chi or qigong could also be appropriate types of exercise to reduce the symptom burden in patients with PV.^35^

There are limited data regarding the effects of targeted exercise information on pain associated with cancer or therapy. Preliminary studies in patients with solid tumors suggest that combined resistance‐endurance training or resistance training alone may help reduce pain.^36^, ^37^ It is possible that patients with PV and chronic pain could also benefit from endurance and/or strength training with blood flow restriction, as has been shown in several patient cohorts.^38^, ^39^

Because of the high proportion of patients with overweight and PV, we suggest that regulation of body weight may be achievable through targeted muscle hypertrophy, which is associated with an increase in lean mass and energy metabolism.^40^ The possibility of additional nutritional counseling should be considered. Patients with obesity and PV should choose joint‐relieving types of exercise such as activities in water or cycling.

In general, considering preferences, two 30–45‐min combined strength‐endurance sessions per week are recommended for sports beginners. This could be done in form of moderate‐to‐vigorous‐intensity circuit training, whereby the load is adjusted progressively. With appropriate exercise selection, consisting of 6–12 exercises for different muscle groups, and the incorporation of endurance exercises such as fast walking, running, cycling, or jumping rope, circuit training is time efficient and comprehensive. Sports‐experienced patients with PV should increase their training volume (frequency and/or time) and aim for at least 150 min/week. Combined resistance‐endurance training can be done independently outdoors or at home using their own body weight and/or small equipment. Outdoors, stairs, benches, or railings could be used for exercises. Patients with PV who prefer group exercise have many options: They could use a sports hall, a gym, or an indoor swimming pool. In principle, patients with PV can participate in all sports, except that in cases of splenomegaly and increased bleeding tendency (e.g., when taking anticoagulants), ball and contact sports or sports with a high risk of falling or injury should be avoided. Because there is no evidence to date on whether free‐weight training is safe and feasible for patients with PV and splenomegaly, low‐injury training on equipment (weight training machines and ergometers) is advised. Patients with PV experiencing skin reactions should refrain from activities in water (chlorinated water) or avoid direct sunlight, depending on the trigger. Generally, adequate sun protection should be used during outdoor activities to prevent melanoma.^4^ Patients with PV who experience headaches or dizziness due to increased blood viscosity should avoid rapid changes in their position during training and ideally should not train alone. Adequate hydration should be ensured.

Because fatigue and concentration problems are independent risk factors for falls in patients with MPN,^23^ older patients with overweight, PV, and these symptoms in particular should consider fall prevention strategies when exercising. Integrating balance exercises into circuit training or performing short independent training sessions with coordination exercises is also possible. Patients with PV should obtain medical approval before starting training, especially if they have obesity or other comorbidities, to avoid adverse events during training.

## 4 DISCUSSION

Here, we present the first study to investigate the level of information and information needs about PA and exercise preferences of patients with PV. Further, novel‐specific PA training recommendations for patients with PV were developed.

A key finding is that patients with PV have a high need for information on PA, regardless on the level of motivation to exercise regularly. The relevance of this topic is strengthened by the relatively young age of the patients with PV at diagnosis, accompanied with a high proportion of employees.^11^ Therefore, PA recommendations should be integrated into the treatment of patients with PV. In this context, treating physicians should inform patients with PV of the true risk of thrombosis or severe bleeding during PA, as fears of such events are barriers to engaging in physical activities.^16^ Considering that the likelihood of anxiety and depression is positively associated with total symptom burden and fatigue, respectively, and negatively associated with QoL,^12^, ^41^ psychological problems should also be systematically assessed and addressed accordingly.^30^

Another finding of this study is that exercise preferences in terms of volume among sports‐inactive patients with PV are significantly below the general exercise recommendations of major health organizations.^6^, ^25^ For example, the American College of Sports Medicine recommends that patients with cancer perform at least 30 min of moderate‐intensity aerobic training three times per week, supplemented by resistance training at least twice a week.^6^ In addition, more than one‐third of patients with PV reported not being action oriented regarding regular sport. This could present challenges to the counseling/treatment team, as behaviors tend to become entrenched over time and are difficult to change.^42^ To implement and maintain changes in exercise behavior, the involvement of a psychologist may be beneficial. In educating patients about the importance and possibilities of PA, as well as in inducing behavioral changes, patients with low educational levels in particular should receive targeted support. Although not the focus of this study, some analyses on the data of the PV cohort reflect the known associations between education/social status and health (morbidity and mortality risks) in the general population.^43^, ^44^, ^45^

Because there have been few (feasibility) studies to date regarding the effects of exercise interventions in patients with PV or MPN,^33^, ^34^, ^46^ we used evidence from studies of patients with other hematological neoplasms or solid tumors to derive the exercise recommendations. In addition, to the best of our knowledge, no study results are available to date regarding whether exercise activities increase skin reactions in patients with PV—for example, due to sweating, friction from clothing, or chlorinated water. Likewise, it is unknown whether sports increase the risk of thrombosis—for example, through increased fluid loss. For this reason, the feasibility, safety, and effectiveness of the novel exercise recommendations for patients with PV should be evaluated and, if necessary, substantiated in subsequent prospective longitudinal studies.

### 4.1 Strengths and limitations

A strength of the study is that despite the low incidence, we recruited a large cohort of patients with PV in German‐speaking regions. Due to the survey design, there are unavoidable limitations that must be considered when interpreting the data. First, as the survey was voluntary and offered online, primarily younger patients with PV and those with an affinity for sports might have participated. Consequently, the proportion of sports‐inactive patients with PV in the population could be higher than the data indicate. Second, the majority of the participants are women, who are known to often report a higher symptom burden.^47^ Because the prevalence of symptoms is consistent with other studies of patients with PV,^11^ potential biases in the results due to unbalanced gender participation might be negligible. Third, we conducted the survey during the COVID‐19 pandemic, so exercise preferences may be biased in terms of the type and location.

## 5 CONCLUSIONS

The current study has shown how important information about PA is for patients with PV. Because these patients benefit from PA in many ways, PA counseling should become an integral part of the treatment plan for patients with PV. This includes regular screening of symptoms. Regarding PA counseling and support for inducing behavioral changes, special attendence should be given to patients with low educational levels. For the first time, we have outlined exercise recommendations based on symptoms to provide specific guidance for patients with PV. Prospective studies evaluating the feasibility, safety, and efficacy of the proposed exercise recommendations are needed to ultimately provide evidence‐based exercise recommendations for patients with PV. In addition, it should be investigated whether providing knowledge about the opportunities and effects of PA leads to a change in the PA behavior of patients with PV.

## AUTHOR CONTRIBUTIONS

**Sabine Felser:** Conceptualization (equal); data curation (equal); formal analysis (equal); funding acquisition (equal); project administration (equal); writing – original draft (lead). **Julia Rogahn:** Formal analysis (equal); writing – original draft (supporting). **Lina Hollenbach:** Formal analysis (equal); writing – original draft (supporting). **Julia Gruen:** Data curation (equal). **Philipp le Coutre:** Data curation (equal); writing – review and editing (equal). **Haifa Kathrin Al‐Ali:** Data curation (equal); writing – review and editing (equal). **Susann Schulze:** Data curation (equal); writing – review and editing (equal). **Lars‐Olof Muegge:** Data curation (equal); writing – review and editing (equal). **Veronika Kraze‐Kliebhahn:** Data curation (equal); writing – review and editing (equal). **Christian Junghanss:** Conceptualization (equal); data curation (equal); supervision (equal); writing – review and editing (equal).

## FUNDING INFORMATION

The study was supported by the East German Study Group Hematology and Oncology (OSHO #97).

## CONFLICT OF INTEREST STATEMENT

The authors declare that they have no competing interests.

## ETHICS STATEMENT

This study was approved by the Ethics Committee of the University of Rostock (A2020‐0274), and registered with the German Registry of Clinical Trials (DRKS00023698).

## Supporting information


Data S1.

Data S2.

## Data Availability

The datasets used and/ or analyzed during the current study are available from the corresponding author upon reasonable request.
