# Supplementary material for: Physical exercise recommendations for patients with polycythemia vera based on preferences identified in a large international patient survey study of the East German Study Group for Hematology and Oncology (OSHO #97)
Source: Cancer Med. 2023 Aug 9;12(17):18235–45. doi: 10.1002/cam4.6413 (PMC10523957; doi:10.1002/cam4.6413)
Supplement: Supplementary file 1 — Data S1. [file CAM4-12-18235-s001.docx]

S1. Member institutions of the East German Hematology and Oncology Study Group (OSHO) participating in the survey

| **Number** | **Institution** |
| --- | --- |
| 1 | Clinic III – Hematology, Oncology and Palliative Care, Rostock University Medical Center |
| 2 | Department of Hematology, Oncology, and Cancer Immunology, Campus Virchow‐Klinikum, Charité‐ Universitätsmedizin Berlin |
| 3 | Krukenberg Cancer Center Halle (Saale), University Hospital |
| 4 | Department of Medicine Clinic II, Hematology, Oncology, Palliative Medicine, Carl-von-Basedow-Klinikum, Merseburg |
| 5 | Department of Internal Medicine III, Heinrich Braun Klinikum Zwickau |
| 6 | Department of Oncology and Hematology, Asklepios Klinik Weissenfels |
| 7 | Oncology and Hematology Practice, Lübsche Straße 146, Wismar |
| 8 | Department of Hematology and Oncology, Leipzig University |
| 9 | Oncology and Hematology Practice, Wismarsche Str. 32, Rostock |
| 10 | Department of Hematology and Oncology, Paul Gerhardt Stift, Wittenberg, Germany |
| 11 | Department of Hematology and Oncology, Klinikum Südstadt Rostock |
| 12 | Oncology and Hematology Practice, Goethestraße 1A, Parchim |
