# Supplementary material for: Physical exercise recommendations for patients with polycythemia vera based on preferences identified in a large international patient survey study of the East German Study Group for Hematology and Oncology (OSHO #97)
Source: Cancer Med. 2023 Aug 9;12(17):18235–45. doi: 10.1002/cam4.6413 (PMC10523957; doi:10.1002/cam4.6413)
Supplement: Supplementary file 2 — Data S2. [file CAM4-12-18235-s002.docx]

S2. Five stages of the transtheoretical model of behavioural change

1. *Intensive physical activity* refers to activities such as jogging, aerobics, speedy cycling, swimming which are performed to increase physical fitness and which usually make you sweat. One speaks of *regular physical activity* if this activity lasts in each case at least 20 minutes and is performed at least on 3 days per week.
2. Are you currently executing *intensive physical activity* on a regular basis for at least 3 days a week for 20 minutes each? Please mark which statement suits you best (please tick only one answer!)

□_1_ No, and I do not intend to start in the next 6 months.

□_2_ No, but I intend to start within the next 6 months.

□_3_ No, but I intend to start within the next 30 days.

□_4_ Yes, but only for less than 6 months.

□_5_ Yes, for more than 6 months.

1. Have you taken any steps in the last 6 months to become physically more active (e.g., bought sports equipment, inquired about an association, walked more)?

□_1_ No □_2_ Yes, what? _____________________________________________________________

Five stages of the transtheoretical Model of behavioral change (SOC) were used to determine the motivation of MPN patients to participate in sports. In order to assign the patients to the SOC, an algorithm was used describing regular sport. By answering the first question, patients were assigned to the stages of precontemplation (stage 1), contemplation (stage 2), action (stage 4) or maintenance (stage 5). Consideration of the second question allowed the assignment to the stage contemplation of preparation (stage 3), however, only if the patients had done something to prepare for physical training (e.g., bought sports equipment) within the last six months. If not, they were assigned to the stage of contemplation (stage 2).

**References:**

Prochaska JO, Redding CA, Evers KE. “The transtheoretical model and stages of change,”. In: Glanz K, Rimer BK, Viswanath K, editors. Health Behavior and Health education: Theory, Research and Practice. Hoboken, NJ: Jossey-Bass (2008). p. 97–122.

Prochaska JO, Marcus BH. “The transtheoretical model: Applications to exercise,”. In: Dishman RK, editor. Advances in exercise adherence. Champaign, IL, England: Human Kinetics Publishers (1994). p. 161–80.
